# Combined incubation of Cadmium, docosahexaenoic and eicosapentaenoic acid results in increased uptake of cadmium and elevated docosapentaenoic acid content in Hepatocytes *in vitro*

**DOI:** 10.1186/s12944-015-0159-2

**Published:** 2015-12-01

**Authors:** Pavla Linhartova, Sabine Sampels

**Affiliations:** Faculty of Fisheries and Protection of Waters, South Bohemian Research Center of Aquaculture and Biodiversity of Hydrocenoses, Institute of Aquaculture and Protection of Waters, University of South Bohemia in Ceske Budejovice, Husova Tř. 458/102, 370 05 České Budějovice, Czech Republic

**Keywords:** DHA, EPA, Fish lipids, Hep G2, Omega-3 fatty acids

## Abstract

**Background:**

Human hepatocellular cells Hep G2 were used to mimic and investigate the effect of the intake of cadmium (Cd^2+^) contaminated fish on cytotoxicity, fatty acid (FA) and phospholipid class composition.

**Methods:**

Cells were incubated with a combination of Cd^2+^ and eicosapentaenoic acid (EPA) and docosahexaenoic acid (DHA) fish specific FA.

**Results:**

We measured a significant increased proportion of EPA and DHA in the treated cells compared to the control line confirming the uptake. While doses of 25 μM DHA showed to be toxic to the cells, repeated short term incubations (2 h) at lower doses resulted in an increased uptake of DHA. The resarzurin assay, evaluating cell viability, showed a significant decrease in cell viability between Cd^2+^ incubation time and, further, the pre-incubation with BSA-FA complex resulted in significantly increased cell viability. On the metabolic level, increased concentrations of EPA and DHA resulted in an increased proportion of docosapentaenoic acid (DPA) which indicated an increased metabolism. Also 24-h combined incubations of 5 μM Cd^2+^ and EPA and DHA showed a significant increase DPA in the total lipid fraction of the cells. In addition, incubation with 5 μM Cd^2+^ for 24 h also decreased the total cardiolipin (CL) fraction from the identified phospholipids.

**Conclusions:**

We confirmed that the applied FA were taken up by the cells. A combination of EPA, DHA and Cd^2+^ influenced lysosomal integrity, cell viability and lipid metabolism in the cells. The most important finding is that DHA and EPA reduced the detrimental effect of Cd^2+^ on cell viability. The exact effects and kinetics behind our observations still need further evaluation.

## Background

Balancing risks and benefits of fish consumption is an intensively discussed public health topic [1–3]. The health benefits from eating fish, partly attributable to omega-3 polyunsaturated fatty acids (n-3 PUFA), are well documented both for prenatal development and adult cardiovascular conditions [4, 5]. Due to their lipophilicity, fish take up and bioaccumulate heavy metals via feed and from the water, which in turn passes up the food chain into the human diet [6]. There have been no studies on the combined effects of FA and Cd^2+^ on cell cytotoxicity.

Cd^2+^ is an environmental pollutant which is taken up with drinking water and food, including seafood and fish. The TDI (daily tolerable intake) for Cd^2+^ was calculated according to the guidelines suggested by the Institute of Standard and Industrial Research of Iran (ISIRI). Based on ISIRI, the tolerable daily intake for Cd^2+^ is 1 μg/day kg of bw (body weight), [7]. Inorganic Cd^2+^ is a human carcinogen [8] and is classified as cancerogenic compound by the International Agency for Research on Cancer (IARC) [9]. Long-term exposure to low concentrations of Cd^2+^ result in accumulation in the liver and the kidneys (kidney cortex) where 30–60 % of ingested Cd^2+^ is deposited [10]. Cd^2+^ compounds have toxic effects on the kidney and are assumed to be neurotoxic. These effects could be due to oxidative stress, but also by different mechanisms that affect the cell membrane composition [11, 12]. Cd^2+^ acts as a catalyst during the formation of reactive oxygen species (ROS).

The long chain n-3 PUFA EPA and DHA have many metabolic functions in animals and humans. Mammals lack the Δ 15 desaturase that is essential for insertion of double bonds at n-3 and hence cannot synthesize the parent FA 18:3 n-3 [13]. In addition it has not been established whether mammals are able to elongate and desaturate 18:3 n-3 towards 20:5 n-3, 22:5 n-3 and 22:6 n-3 in significant amounts [14–16]. Therefore, these FA are regarded as essential for humans and they need to be included in the diet. They are vital nutrients and precursors of several metabolites, which are potent lipid mediators, known to be beneficial in the prevention and treatment of several diseases [17]. Fish are an important source of these FA and hence the consumption of fish (200 g portion) at least twice a week is recommended by several health organizations including FAO/WHO [18]. In the same report, the negative effects of eating contaminated fish are out weight against the benefits of the FA. However, it is unclear which exact mechanisms are affected by cadmium intake in relation to lipid metabolism and oxidative stress when it is simultaneously taken up with the long chain n-3 FA from fish.

The aim of the present study was therefore to evaluate the combined effects of the nutritional essential FA EPA and DHA from fish and Cd^2+^. Our hypothesis took two aspects into account: Cd^2+^ is known to create oxidative stress in the cells, so one of the toxic effects could be the oxidation of important membrane or organelle FA or phospholipids, subsequently leading to cellular dysfunction or apoptosis. If contaminated fish are ingested, the long chain n-3 FA from the fish, which are the FA most easily oxidized, could prevent or decrease the impact of Cd^2+^ either by replacing the oxidized FA, or conversely by increasing the oxidative stress and thereby worsen the effects of the Cd^2+^. Hepatocytes were selected for the investigation because the liver is a target organ for Cd^2+^. A combination of DHA/EPA as 2/1 reflects the typical ratio in many fatty fish, common carp (*Cyprinus carpio*) for example, which is traditionally consumed in Czech Republic. In order to mimic the uptake of contaminated fish, we investigated the metabolic effects of cadmium (Cd^2+^) on the cell line Hep G2 in combination with the long chain n-3 PUFA, eicosapentaenoic acid (EPA; 20:5 n-3) and docosahexaenoic acid (DHA; 22:6 n-3) which are typical for fish [19, 20].

## Methods

### Caution

Inorganic cadmium chloride (CdCl_2,_ Cd^2+^) is classified as a human carcinogen [21] it is hazardous, or potentially hazardous and should be handled with care.

### Chemicals

Eicosapentaenoic acid (EPA) and docosahexaenoic acid (DHA) supplied from Biochrom and Sigma-Aldrich (Berlin, Germany), were diluted in extra pure 98 % ethanol and bovine serum albumin (BSA) before transfer experiments. Hydrogen peroxide solution (30 %, Suprapurs) and nitric acid (65 %, Suprapur) were products of Merck (Darmstadt, Germany). Cadmium chloride was obtained from Aldrich, Germany. All other pro-analysis chemicals were obtained from Sigma-Aldrich (Steinheim, Germany) and Merck (Darmstadt, Germany). Trypsin, penicillin and streptomycin solutions were products of Sigma (Deisenhofen, Germany). Moreover, the culture dishes and the culture medium (MEM) for Hep G2 cells were obtained from Biochrom (Berlin, Germany).

#### Cell culture

Human hepatocellular cells (Hep G2, ATCC, No. HB-8065) were purchased from the American Type Culture Collection (ATCC, Manassas, V C, USA). Hep G2 cells were grown as a monolayer in culture dishes in Minimum Essential Medium Eagle (MEM) supplemented with FCS (10 %, v/v), non-essential amino acids (1 %, v/v), glutamine (2 mM), penicillin (100 U/mL) and streptomycin (100 μg/mL). The Hep G2 cultures were incubated at 37 °C with 5 % CO_2_ in air with 100 % humidity. Cells were passaged every 3 days. The amount of 1.5 million cells were seeded on 10 cm (in diameter) sterile Petri dishes in 10 mL of sterile culture medium (MEM). Suspensions of Hep G2 cells were produced from confluent cultures using trypsin/EDTA solution. Before the transfer experiments, cells were three times sub-cultured to achieve a stable phenotype. For the transfer experiments, cells were seeded at a density of 66.7 cells per μL for 96 well plates and 1.5 Mio (million) per normal petri dishes (10 cm in diameter). Seeded Hep G2 were cultured for 24 h and 37 °C and subsequently prepared for pre-incubations and post-incubations with FA and Cd^2+^. Hep G2 cells where used from passage Nr. 20 at least 3–4 weeks (till passage Nr. 35). Hep G2 cells can be used from the third passage to Nr. 130. Hep G2 cells were incubated with Cd^2+^ only, with Cd^2+^ and with BSA solved in PBS in order to evaluate if the BSA alone would have any effects and finally with Cd^2+^ and the FA (EPA + DHA) as a BSA-FA complex.

#### EPA and DHA pre-incubations

Before incubation fresh stock solutions of FA diluted in extra pure 98 % EtOH were defrosted. The BSA-FA complex was prepared as follows: BSA was dissolved in PBS (phosphate buffer saline). EPA and DHA were dissolved in extra pure EtOH to a final volume of 50 μL. Then 20 μL of EPA and DHA solutions were added to 1 ml of a mixture of 0.1 M NaOH and BSA solution (1/5; v/v) each. The two solutions of FA were then combined and the pH was adjusted to 7.1 using 0.1 M HCl. After testing concentrations from 1–50 μM EPA and from 2–100 μM DHA, we chose concentrations for pre-incubations of liver human cells with 5 μM EPA+ 10 μM DHA for 2–48 h without changing the cell culture medium (MEM).

#### Cd^2+^ post-incubations

Fresh stock solutions of Cd^2+^ diluted in distilled sterile water (ddH_2_O) were prepared before the transfer experiments. Hep G2 liver cells were post-incubated for 24 h or 48 h with Cd^2+^ stock solution with changing the cell culture medium (MEM). The range of Cd^2+^ concentrations were used from min. 0.25 μM to max. 20 μM.

#### Cytotoxicity and lysosomal integrity

The neutral red assay was used to assess the impact of the heavy metal on lysosomal integrity [22] using a plate reader (Invinite 200Pro, Tecan Group Ltd., Mannedorf, Switzerland). The levels of IC_50_ and IC_70_ were measured so as to indicate the percentage of control in the parameter of lysosomal integrity.

#### Cytotoxicity and cellular viability

Cellular viability, was measured with the resarzurin uptake assay [23] using a plate reader (Invinite 200Pro, Tecan Group Ltd., Mannedorf, Switzerland). The levels of IC_50_ and IC_70_ were measured so as to indicate percentage of control in the parameter of cell viability.

### Pelleting of Hep G2 cells

Cells were seeded in number of 1.5 million cells per Petri dish to 10 mL MEM sterile culture medium. Seeded Hep G2 were cultured and treated with FA as a BSA-FA complex and Cd^2+^ for the appropriate times as described above. Subsequently cells were pelleted by trypsinising and centrifuging several times in PBS/FKS solution and frozen on −80 °C.

### Fatty acid composition and phospholipid classes

The pelleted cells were re-suspended in buffer and the total lipids were extracted from cells according to Hara and Radin [24]. From these extracts, the composition of major lipid classes as well as phospholipid classes were evaluated via automated high performance thin layer chromatography (HPTLC), [24, 25]. For analyses of FA composition, methylation of total lipids was performed by using a combination of NaOH and BF_3_ according to Appelqvist [26]. FA composition was then analyzed by GC on a BPX-70 50 m fused silica capillary column (id. 0.22 mm, 0.25 μm film thickness, SGE, USA) as described Sampels et al. [27]. Identification of FA and phospholipid classes was done by commercially available external standards (Nu-Check Prep, Inc. Elysian USA; Avanti Polar Lipids, Inc., Alabama, USA) quantification of the FA was done by internal standard (C 21:0, Nu-Check Prep, Inc. Elysian USA). Total fat and phospholipids and FA as % of total identified per million cells in μg were evaluated.

### Determination of cadmium uptake

The content of cadmium chloride was analyzed via ICP-MS as described earlier [28]. Quantification was performed with authentic standards. Cd^2+^ concentration in μM in pelleted cells was analyzed.

### Statistical analysis

All analyses were conducted in triplicate or quadruplicate. Normality and homogeneity of dispersions of studied values and comparisons were made by analysis of variance (two factorial ANOVA; factors: Cd^2+^ and FA) with subsequent post hoc Tukey’s honest significant difference (HSD) test. The values were expressed as means ± SD (*n* = 3). All analyses were performed at a significance level of *p <* 0.05 using STATISTICA 9.0 for Windows.

## Results

### FA concentrations suitable for incubation trials

**Fig. 1:**
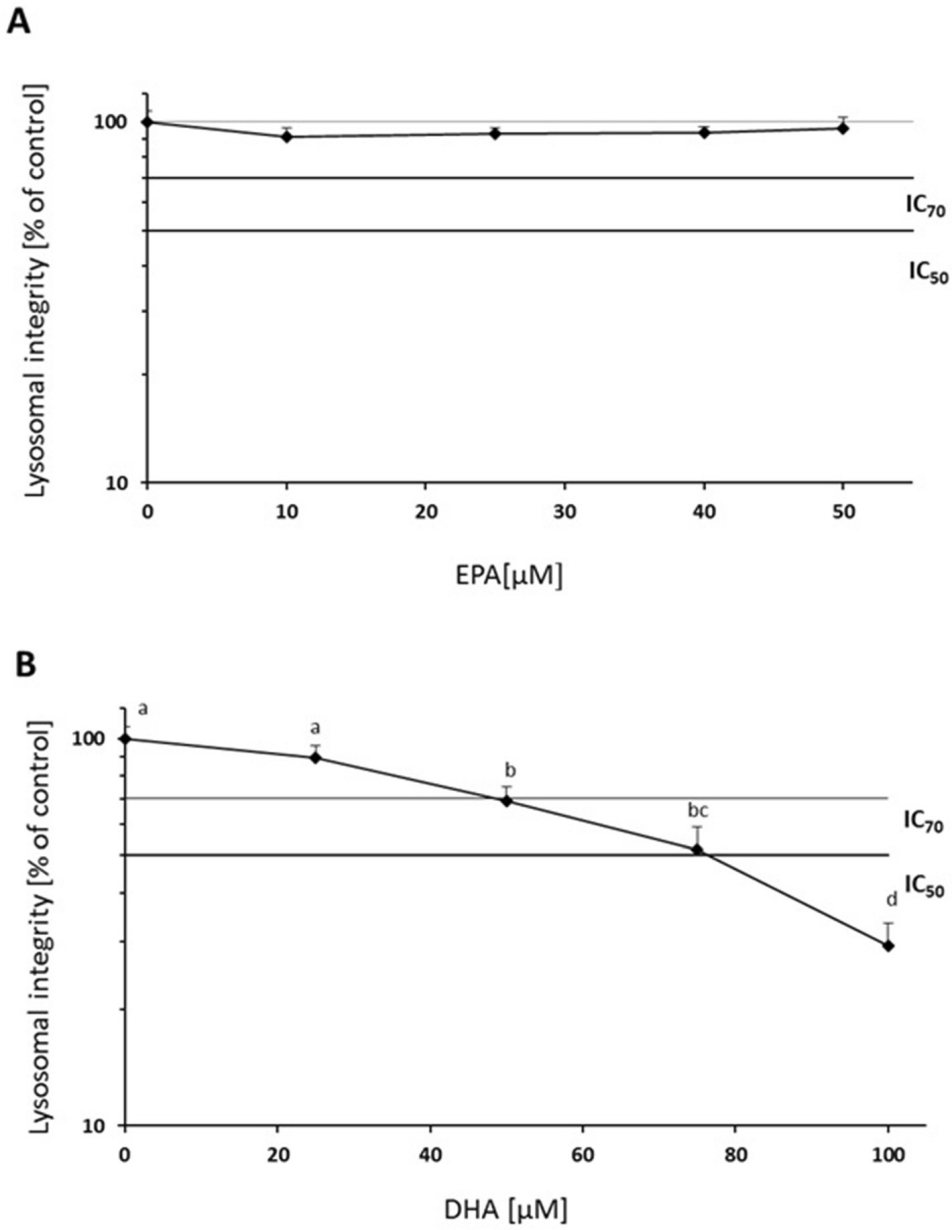
Lysosomal integrity after *in vitro* pre-incubations of Hep G2 for 24 h with EPA (**a**) at nominal concentrations of 10, 25, 40 and 50 μM and DHA (**b**) at nominal concentrations of 25, 50, 75 and 100 μM compared to control cells. Data are presented as means ± SD, *n* = 3. Different letters denote significant difference between treatments (ANOVA, *p <* 0.05)

**Fig. 2:**
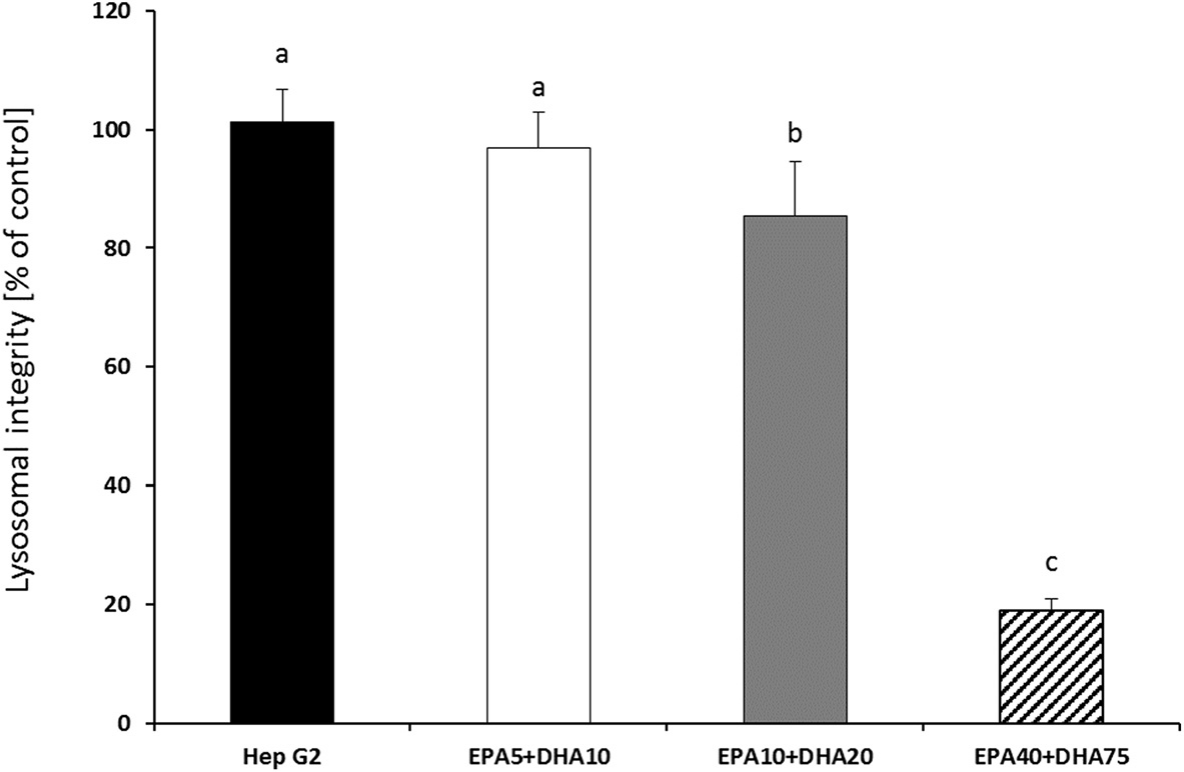
Lysosomal integrity after *in vitro* pre-incubations of Hep G2 for 24 h with combined EPA+ DHA. Nominal concentrations of EPA5 + DHA10 µM, EPA10 + DHA20 μM and EPA40 + DHA75 μM compare to control cell line (Hep G2). Data are presented as means ± SD, *n* = 3. Superscript letters indicate significant differences between treatments (ANOVA, *p <* 0.05)

In order to establish suitable incubation concentrations of FA, we first incubated the cells with the individual FA ranging for EPA from 1 μM to 50 μM (Fig. 1a) and for DHA from 2 μM to 100 μM (Fig. 1b), respectively. For EPA, the highest 50 μM concentration was above toxic effects for the cells (Fig. 1a). Neither the level of IC_50_, nor level of the IC_70_ was reached. No significant change on cell growth measured by lysosomal integrity was found for this FA. For DHA, significant changes were found only between control line (Hep G2) and cells treated with 25 μM DHA and higher DHA concentrations showed significant toxic effects on the cells (Fig. 1b). A significantly negative effect of DHA on Hep G2 cell growth was observed after *in vitro* for 24 h at concentrations 50 μM DHA (IC_70_) and the IC_50_ was reached at the level of 76 μM DHA (Fig. 1b). Furthermore, the cells showed only 29.3 % vitality at a level of 100 μM DHA (Fig 1b). In a second step we evaluated the effects of the incubation with a combination of EPA and DHA. The combination of 40 μM EPA+ 75 μM DHA resulted in a cell viability significantly below 50 % (18.9 % viable cells), while the combination of 10 μM EPA+ 20 μM DHA, was above the level IC_70_ (85.3 % vital cells) (Fig. 2), but was significantly lower compared to the control cells without added FA. As the level of DHA was still too high, we decreased the concentration to a combination of 5 μM EPA+ 10 μM DHA (Fig. 2). With these concentrations a cell viability of 96.9 % (EPA5 + DHA10) was reached. No cytotoxic effects of FA on cell growth in this combination with control line were found.

### Cell viability

**Fig. 3:**
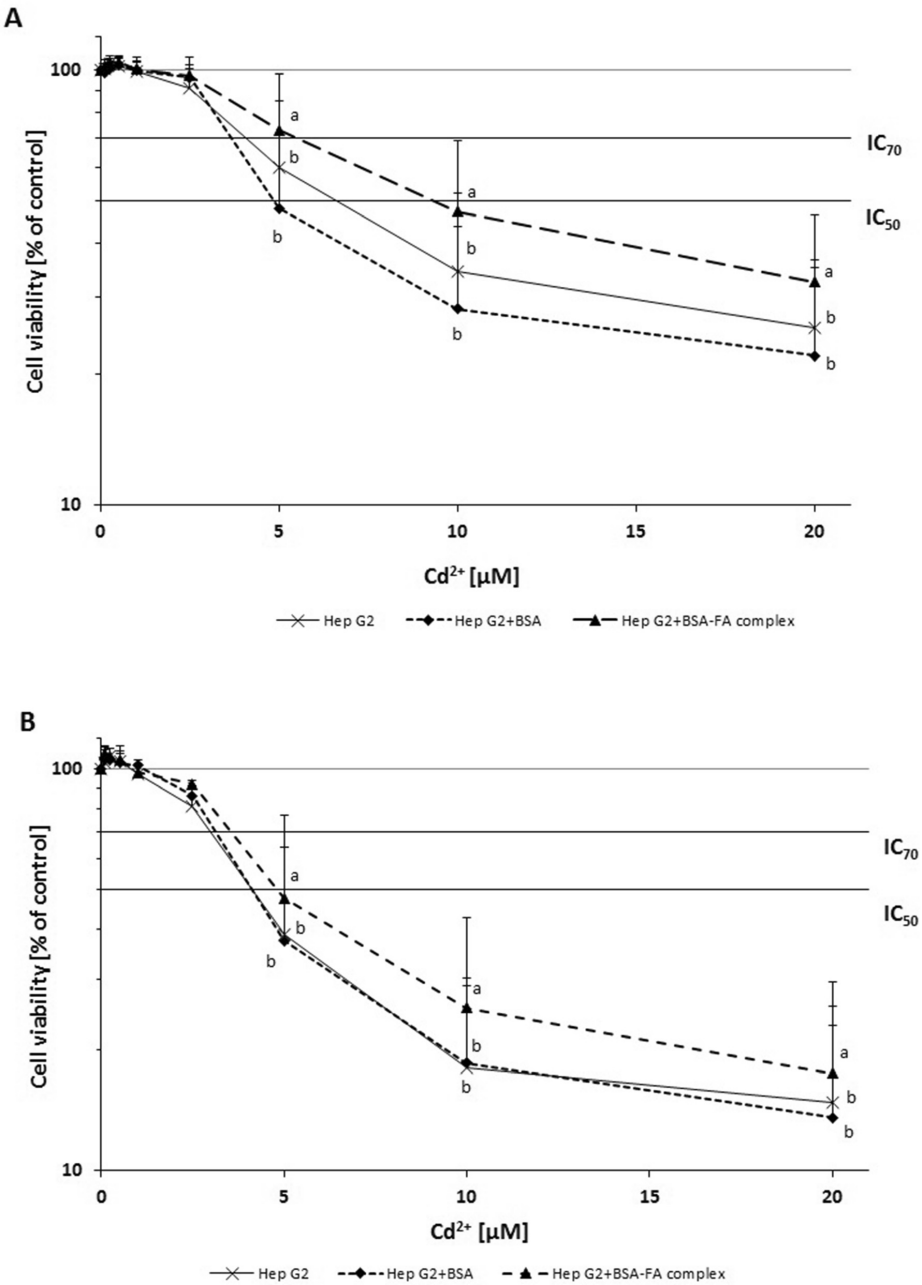
Cell viability after *in vitro* pre-incubations of Hep G2 for 24 h with 5 μM EPA+ 10 μM DHA dissolved as BSA complex and 24 h (**a**) and 48 h (**b**) post-incubations with Cd^2+^ at nominal concentrations of 0.25, 0.5, 1, 2.5, 5, 10, 20 μM with changing culture (MEM) medium. Data are presented as means ± SD, *n* = 3. Different letters denote significant differences between treatments (two factorial, ANOVA, (FA, Cd^2+^), *p* < 0.05)

The resarzurin assay, evaluating cell viability, showed a significant correlation between Cd^2+^ incubation time and decreasing cell viability; an IC_50_ value of 6.6 μM and IC_70_ of 4 μM were measured after 24 h (Fig. 3a: Hep G2) and an IC_50_ value of 4.1 μM and IC_70_ of 3 μM after 48 h (Fig. 3b: Hep G2). Cell viability was significantly different in comparison to the control cells at 5 μM Cd^2+^ for both incubation times (Fig. 3ab).

Pre-incubation with the BSA-FA complex resulted in significantly increased cell viability at concentrations starting at 5.5 μM Cd^2+^ (IC_70_) for FA group in comparison with only 4 μM Cd^2+^ for control-Hep G2 (IC_70_) and only 3.6 μM Cd^2+^ (IC_70_: Hep G2 + BSA), after 24 h post-incubations with Cd^2+^ (Fig. 3a). No effects of BSA dissolved in PBS alone incubated with the cells (Hep G2 + BSA) were found compare to control (Hep G2), which demonstrated that the BSA can be used without affecting cell growth (Fig. 3ab).

### Hep G2 lipid composition: phospholipids and fatty acids

**Fig. 4:**
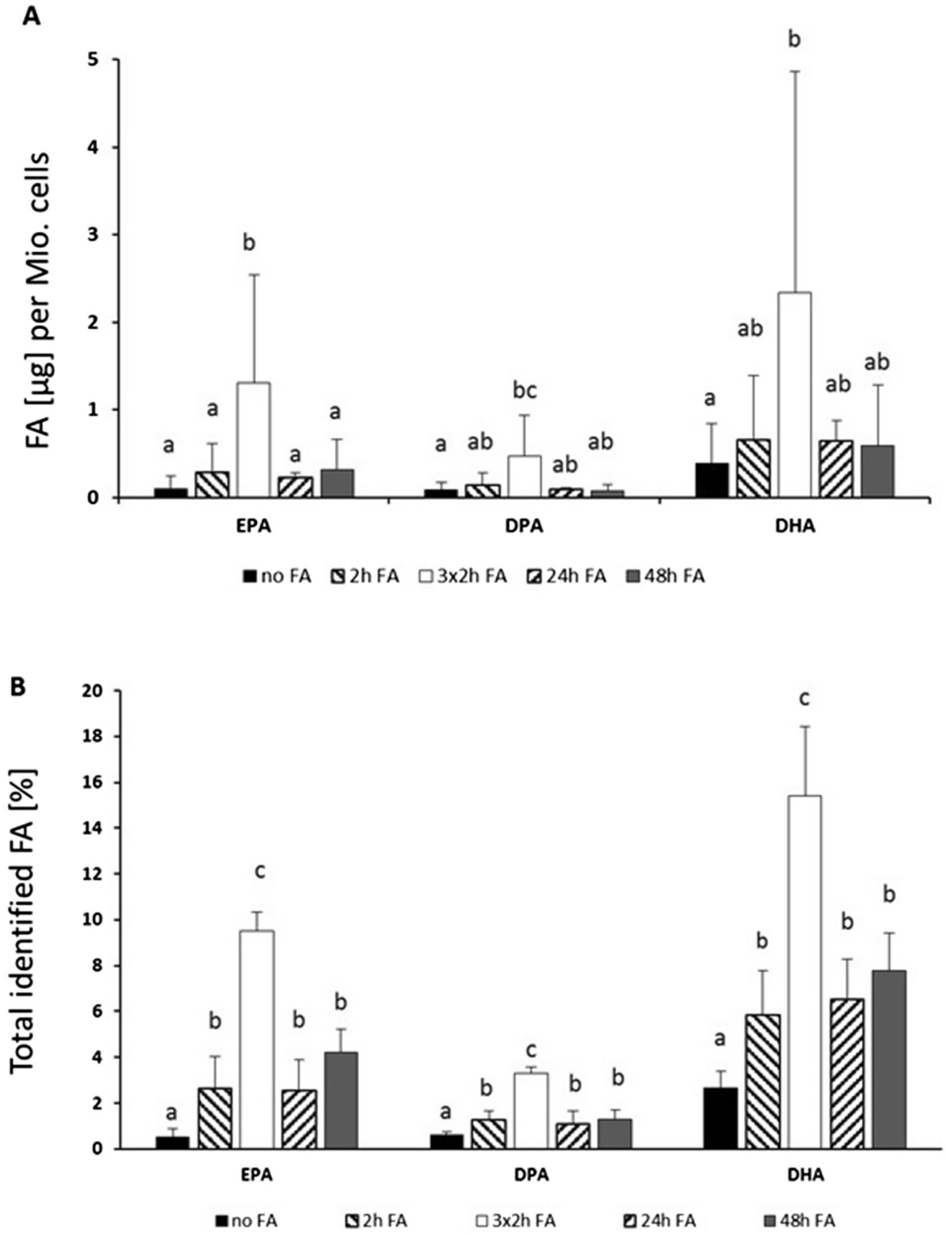
Content of EPA, DPAn-3, DHA and total amount of FA after *in vitro* incubations of Hep G2 for 2, 3x2, 24 and 48 h with 5 μM EPA+ 10 μM DHA as BSA complex and different subsequent time of cell growth (30, 48 and 96 h), presented as μg of FA per Mio. cells (**a**) and total identified FA (%) content (**b**). ‘No FA’ means cells were only incubated with BSA as control. Data are presented as means ± SD, *n* = 3. Different letters denote significant differences of the respective FA between incubation times (ANOVA, *p <* 0.05)

Table 1 shows the contents of the long chain omega3-FA (EPA, DPA, DHA) in the cells with or without incubation of the FA for 24 h. A significant increase in the cells of both EPA and DHA was shown after the incubation (Fig. 4a and b). We demonstrated that after only two hours the maximum uptake of FA (Fig. 4b) was reached and there were no significant difference between 2, 24 or 48 h of FA incubation per million cells in Fig. 4a. Further we showed that repeated incubations were more effective. This was probably due to the totally increased substrate. After three replicated 2-h incubations with EPA or DHA (total 6 h of FA incubation of the cells with changing the medium every 2 h) proportions of FA in the cells were significantly increased compared to all other treatments and without toxic effects. When expressed as percentage of total fat, the increase of DPA was also significant after the incubation with FA; this suggests a synthesis from EPA toward DHA (Fig. 4b). When expressed as μg of fat per million (Mio.) cells, the increase of DPA was significant for only the 3 × 2-h incubation, which supports the hypothesis that an increased proportion of EPA will lead to an increased metabolism towards DHA (Fig. 4a). The level of EPA reached 1.31 μg per Mio. cells and the DHA level was almost eight times higher (2.33 μg per Mio. cells) compared to untreated cells (Fig. 4a). Moreover, EPA increased to 9.5 % and DPA to 3.3 % and the DHA level was almost three times higher (15.4 % of total identified FA than the untreated cells (Fig. 4b). In the control line, without FA incubation, both EPA and DPA showed levels of 0.6 % and DHA 2.7 % of total identified FA (Fig. 4b). Even though uptake had reached the maximum after 2 h, for practical reasons we decided to use 24-h pre-incubations for the subsequent FA and Cd^2+^ experiments.

**Table 1:** Fatty acid content in Hep G2 cells

μg FA per Mio cells	BSA-control no FA	BSA-FA (24 h)	BSA-control no FA Cd5 (24 h)	BSA-FA (24 h) Cd5 (24 h)
C20:5n-3	0.09 ± 0^a^	0.37 ± 0.09^b^	0.14 ± 0.05^a^	0.59 ± 0.14^b^
C22:5 n-3	0.10 ± 0.01^a^	0.16 ± 0.03^ab^	0.17 ± 0.07^ab^	0.31 ± 0.06^b^
C22:6n-3	0.40 ± 0.06^a^	0.84 ± 0.08^b^	0.57 ± 0.22^a^	1.23 ± 0.19^b^
SFA	5.61 ± 0.98	5.47 ± 0.66	7.24 ± 3.20	8.38 ± 2.19
MUFA	8.19 + 0.98^a^	6.02 ± 0.33^b^	8.65 ± 2.88^ab^	7.48 ± 1.30^b^
PUFA	2,00 ± 0.29^a^	2.60 ± 0.28^b^	2.84 + 1.08^ab^	4.01 ± 0.44^b^
n-3	0.59 ± 0.07^a^	1.38 ± 0.10^b^	0.88 ± 0.34^a^	2.13 ± 0.23^b^
n-6	1.36 ± 0.26^ab^	1,20 ± 0,19^b^	1.94 ± 0.76^a^	1.81 ± 0.30^ab^
n-6/n-3	2.28 ± 0.18^a^	0.87 ± 0.08^b^	2.21 ± 0.02^a^	0.85 ± 0.08^b^

FA content (% of total identified) in Hep G2 cells incubated with or without FA (24 h) and subsequently with or without 5 μM Cd^2+^ for 24 h. ‘No FA’ means cells were only incubated with BSA as control. Data are presented as means ± SD, *n* = 3. Different letters denote significant differences between treatments (two factorial ANOVA, (FA, Cd^2+^), *p <* 0.05)

*Abbreviations*: *PC* L a-Phosphatidylcholine, *CL* Cardiolipin, *PA* L-a-Phosphatidic acid, and *EA* Phosphatidylethanolamine

Incubation with Cd^2+^ did not influence the content of EPA or DHA, (Table 1). However the subsequent incubation of the cells with the FA and 5 μM Cd^2+^ resulted in a significant increase of DPA.

**Fig. 5:**
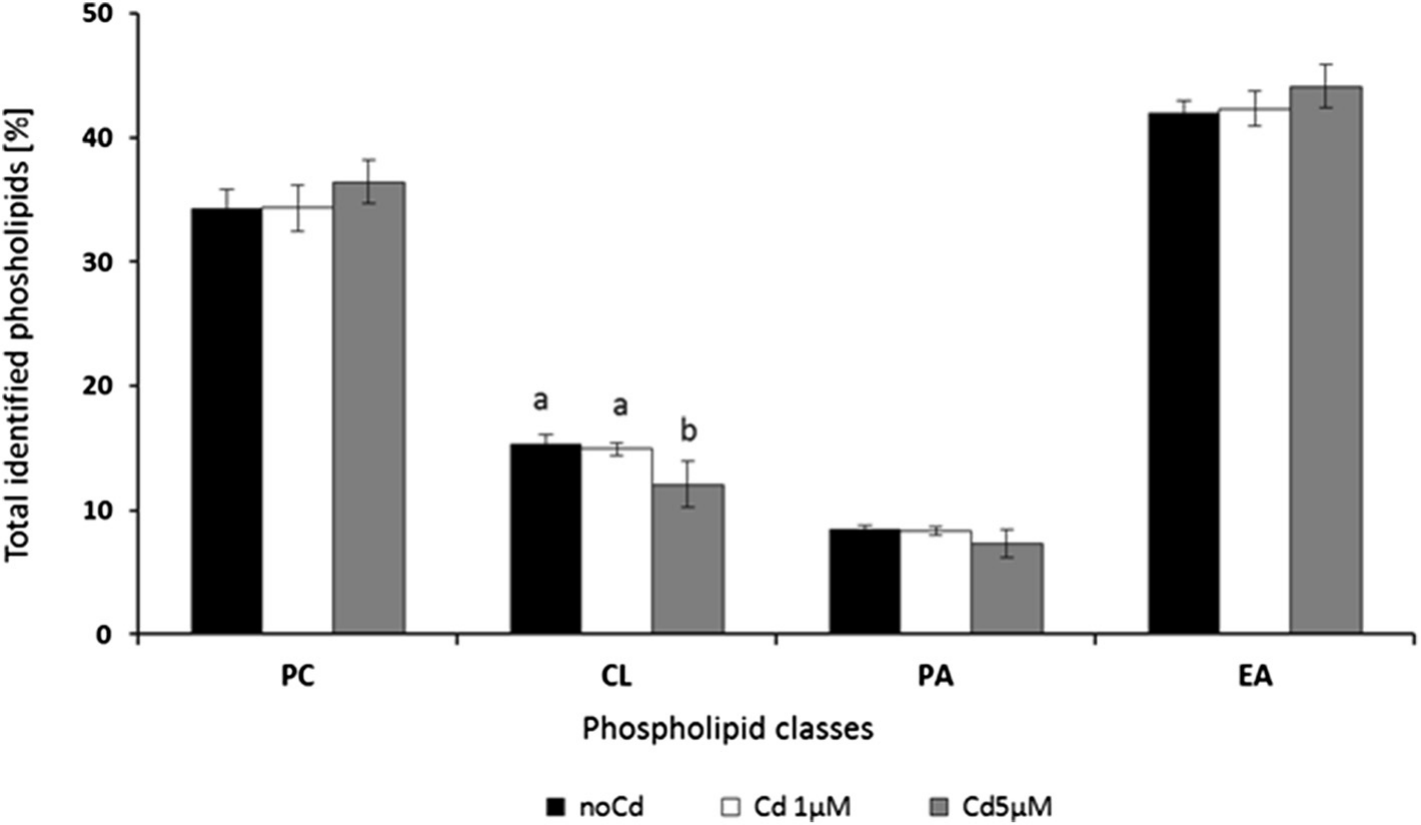
Phospholipid class composition presented as percentage of total identified (means ± SD, *n* = 3) after *in vitro* pre-incubations of Hep G2 for 24 h with 5 μM EPA+ 10 μM DHA dissolved as BSA complex and 24 h post-incubations with/without Cd^2+^ at nominal concentrations of 1 and 5 μM with changing culture (MEM) medium. Data are presented as means ± SD, *n* = 3. Different letters denote significant differences between treatments (two factorial ANOVA, (FA, Cd^2+^), *p <* 0.05)

There were no significant differences in phospholipid composition of the cells related to the pre-incubation with FA. Therefore, the data were combined to facilitate statistical evaluation. Figure 5 shows the phospholipid class composition in the Hep G2 after the various incubations. The phospholipids cardiolipin (CL) decreased significantly after incubation with 5 μM Cd^2+^ for 24 h. At this level of cadmium, only 12.1 % of CL were detected in cells compared to 14.9 % at 1 μM Cd^2+^ and 15.4 % without any cadmium treatment (Fig. 5). The decrease of CL between 1 μM and 5 μM incubations was significant (*p* < 0.05).

### Uptake of Cd in relationship with FA

**Fig. 6:**
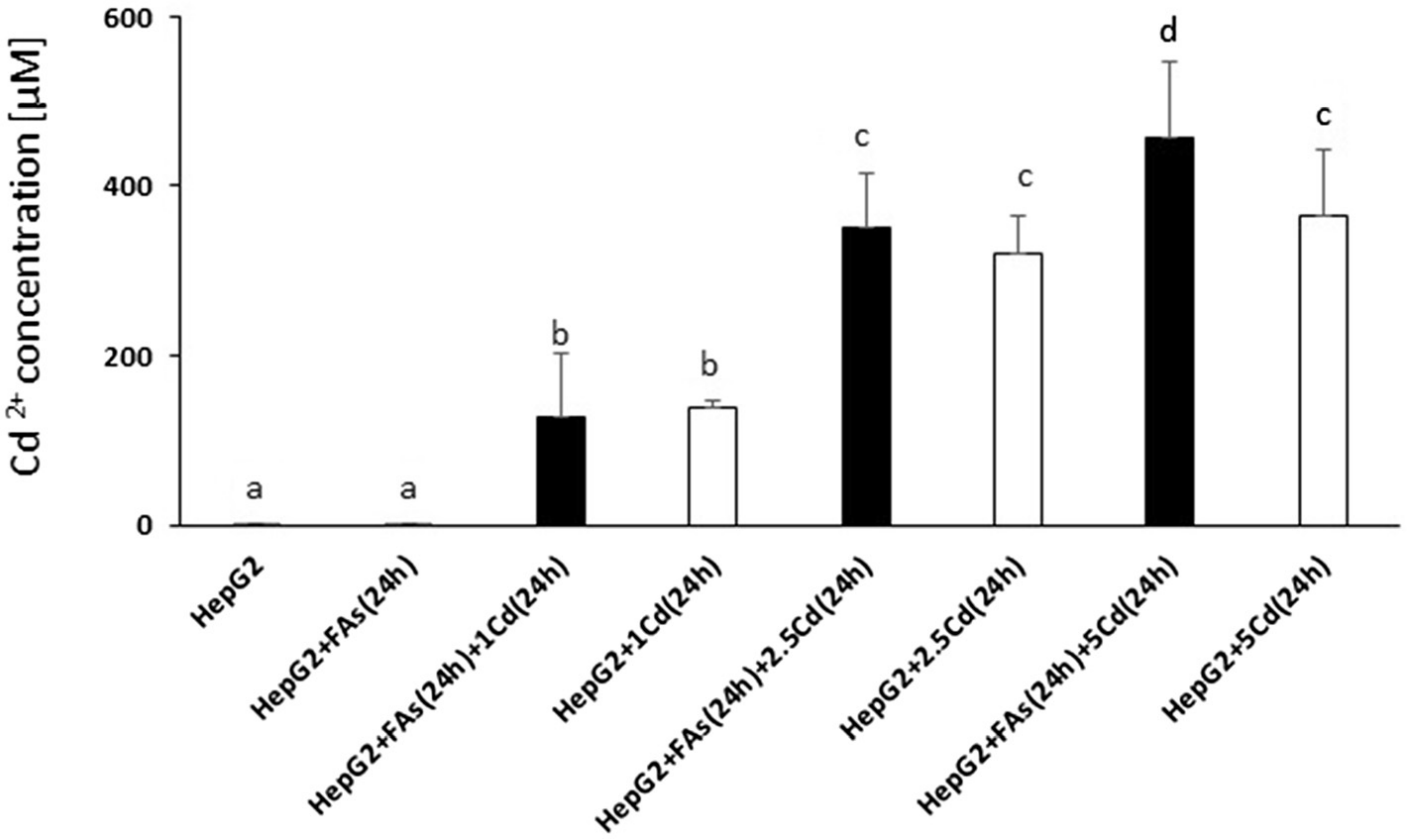
Uptake of FA (24 h) and Cd^2+^ (1, 2.5 and 5 μM) for 24 h after *in vitro* incubations on Hep G2 cells with changing culture (MEM) medium. ‘No FA’ means cells were only incubated with BSA as control. Data are presented as means ± SD, *n* = 3. Different letters denote significant differences between treatments (two factorial ANOVA, (FA, Cd^2+^), *p <* 0.05)

Figure 6 shows the result from Cd uptake, which was verified by ICP-MS. Three different concentrations of Cd (1, 2.5 and 5 μM) were tested. One-half of pelleted cells was pre-incubated with FA for 24 h and all groups where then post-incubated with Cd^2+^ for the same time. The group incubated with the highest 5 μM Cd^2+^ and FA (5 μM EPA + 10 μM DHA), had a significantly higher uptake of cadmium chloride (457.6 365.8 μM), compared to the group not treated with FA (365.8 μM μM Cd^2+^).

## Discussion

In the present study we used a combination of EPA + DHA in a ratio 1:2 which is a proportion of FA typical for fatty fish such as common carp (*Cyprinus carpio*); carps are available on the local market year round [29]. Despite that fact that 60 μM DHA has been considered as physiological relevant [30], we found that the IC_70_ level had been already reached at 50 μM DHA. Our findings are in agreement with earlier results that DHA has cytotoxic effects on cancerogenic cells; similar toxic effects of DHA on cancerogenic neuroblastoma cells but not on non-transformed nervous tissue have been reported by Lindskog et al. [31]. These authors concluded that DHA counteracted cancer by causing apoptosis in the cancer cells. Juaudzus et al. [32] also showed that relatively high doses of DHA did not affect healthy cells. In the cell lines MIA PaCa-2, PANC-1 and CFPAC (human pancreatic cell lines), EPA was also shown to have an inhibitory effect of cell growth (IC_50_ 2.5-5 μM), [33]. In contrast, we found cytotoxic effects of DHA but not of EPA at concentrations of 50 μM. The mechanisms by which DHA acts differentially on cancerogenic versus normal healthy cells are still under discussion. In their review [34] describes the main mechanism to oxidative stress created by oxidation compounds derived from DHA. Due to their increased metabolism, cancer cells have increased natural levels of ROS and additional ROS can then not be counteracted as well as in healthy cells where the antioxidant mechanisms are still intact.

According to our results in the present setting, 5 μM EPA+ 10 μM DHA were the most suitable incubation concentrations for hepatocellular human liver cells (Hep G2; ATCC). We showed that the effect of EPA and DHA on the cells seems to be different, which should be evaluated further.

We showed that the incubated FA was taken up by the cells (Table 1), however, we found an increase of those FA only in the total lipid fraction (Table 1) and not in the subcellular lipid fractions (data not shown), indicating that an incubation time up to 48 h is probably too short for the FA to be incorporated into the cell structure.

When evaluating the incubation times, the maximum uptake already had been reached after only 2 h incubation with FA. After this, the proportion of the used FA did not significantly further. In contrast, Obermeier et al. [35] showed that the maximum levels of incorporation of AA, EPA, and DHA into U937 cell (human leukemic monocyte lymphoma cell line) was reached after 8 h. This finding suggests that different cell types might have different time optima for the uptake. Consequently, we decided to use 24-h incubation times for improved practical handling and to ensure a good repeatability.

In line with our results, Fujiyamafujiwara et al. [36] also found that incubation of Hep G2 cells with EPA resulted in a dose-dependent incorporation of EPA and a metabolism towards DPA but not DHA. The authors also found an increase of DHA, but a lower dose dependency, plus an increased amount of DPA in the cells after incubation with DHA, suggesting that DHA was ß-oxidized into DPA. These findings support our hypothesises that an increased proportion of substrate, in our case EPA, results in increased synthesis of the longer chain FA as well as a higher ß-oxidation of increased amounts of DHA into DPA.

In addition, we showed that repeated incubations are more effective. This is probably due to the totally increased substrate. In this way it seems to be possible to load the cells with greater amounts of EPA and DHA without the toxic effects caused by higher concentrated doses. This finding could be useful in future studies of the effects of elevated PUFA levels. On a metabolic level, the increased substrate also seems to result in a higher metabolism from EPA towards DPA and possibly DHA.

One of our hypotheses was that Cd^2+^ could cause a change in the cell lipid composition resulting in dysfunction and finally apoptosis. The results from the resazurin assay, with a significant correlation between Cd^2+^ incubation time and decreasing cell viability concurred with this, as the resarzurin method is based on the fact that normal viable and healthy cells reduce the blue resazurin to the pink resorfin. It is known that resazurin is effectively reduced in the mitochondria, making it useful also to assess mitochondrial metabolic activity. Hence we think that Cd^2+^ affects the mitochondrial functions. A reason for this could be oxidation of essential membrane lipids. An indication for this is that the highest used concentration of Cd^2+^ resulted in also decreased levels of cardiolipin (CL) in our study. CL is exclusively localized in the inner mitochondrial membrane and important for mitochondrial membrane functionality [37]. CL is especially sensitive to oxidation due to the high content including DHA [34]. Increased oxidation and subsequent decrease of CL has been connected to apoptosis [34] which is also inferred by our study.

In conclusion, our results, showed that a decrease in the proportion of CL together with a decreased cell viability, indicating that Cd^2+^ has an effect on cellular lipid composition and mitochondrial function.

Interestingly, the co-incubation of FA and Cd^2+^ resulted in a significantly higher uptake of Cd^2+^ at the highest concentrations of Cd^2+^ (5 μM), while the uptake seemed to reach a maximum level at 2.5 μM (Fig 6). This finding is in contrast to a study by Nostbakken et al.*,* [38] evaluating the effect of EPA and DHA on methyl mercury (MeHg) uptake. In that study DHA decreased the uptake of MeHg in HEK293. In line with our results, DHA increased the uptake of the heavy metal and MeHg induced apoptosis in ASK. However, in the same study, EPA had an opposing effect and decreased the uptake of MeHg. Since we used a combination of these FA in our study, this could also mean that we had mixed increasing and decreasing effects on the uptake of Cd^2+^. However, this hypothesis needs further evaluation.

Another interessting result from our study was that the combined incubation of the FA and Cd^2+^ at the highest level increased the proportion od DPA in the cells. This indicates either an upregulated metabolism from EPA towards the longer chain products or an increased β-oxidation from DHA due to energy needs of the cells or oxidative stress. We hypothecize that Cd^2+^ has an enhancing effect on these processes. Therefore, the next step should be to evaluate various levels of oxidation and antioxidant response on the cells as well as the protein expression of the related elongases.

## Conclusion

The findings of the present study showed that the applied FA were taken up by the cells and that subsequent incubation with Cd^2+^ did not decrease the contents of EPA and DHA. However, as a possible adverse effect, the combined incubation of FA and Cd^2+^ resulted in a significantly increased uptake of Cd^2+^ at the highest used levels. The possible toxic effects of this findings in vivo should be evaluated.

It should be highlighted that both FA (EPA and DHA) reduced the detrimental effect of Cd^2+^ on cell viability, which is the most relevant finding of present study with potentially important implications regarding fish consumption.

Further, the combined incubation of EPA and DHA and Cd^2+^ increased the levels of DPA in the total lipid content of the cells, which could either be a sign of increased metabolism from EPA to DPA or increased ß-oxidation from DHA to DPA induced by the cadmium. We also found that incubation with Cd^2+^ decreased CL, an essential phospholipid class in the mitochondria, indicating that part of the toxic effects of cadmium is related to mitochondria dysfunction. However, the reason for the increased DPA values needs to be investigated as well as the general oxidative stress parameters, which will be the subject of our further work.

## Abbreviations

AA: arachidonic acid (C20:4 n-6)
BSA: bovine serum albumin
BSA-FA: bovine serum albumin complex with fatty acids
Cd^2+^: cadmium chloride
CL: cardiolipin
DHA: docosahexaenioc acid (C22:6 n-3)
DPA: docosapentaenoic acid (C22:5 n-3)
EA: phosphatidylethanolamine
EPA: eicosapentaenoic acid (C20:5 n-3)
FA: fatty acids
MEM: Minimum Essential Medium Eagle
PA: L-α-phosphatidic acid
PBS: phosphate saline buffer
PC: L α-phosphatidylcholine
PUFA: polyunsaturated fatty acids.
